# The efficacy and safety of proton-pump inhibitors in treating patients with non-erosive reflux disease: a network meta-analysis

**DOI:** 10.1038/srep32126

**Published:** 2016-09-01

**Authors:** Lingxiao Chen, Yujie Chen, Bo Li

**Affiliations:** 1 Tianjin Medical University, Tianjin, China; 2 Department of Oncology, Tianjin Medical University General Hospital, Tianjin, China; 3 Department of Clinical Epidemiology and Evidence-based Medicine, Beijing Hospital of Traditional Chinese Medicine, affiliated with Capital Medical University, Beijing Institute of Traditional Chinese Medicine, Beijing, China

## Abstract

Proton-pump inhibitors (PPIs) have been proved as safe and effective ways to treat patients with non-erosive reflux disease (NERD). However, less is known about the comparisons among different PPIs and their best dosage. We aimed to synthesize the available evidence through network meta-analysis to investigate the efficacy and safety of different PPIs in treating patients with NERD. Fifteen studies with 6309 patients were included in the meta-analyses. For the rate of symptomatic relief, compared with control groups, all interventions except rabeprazole 5 mg significantly increased rate of symptomatic relief. Among the comparisons of different interventions, omeprazole 20 mg group was associated with a higher rate of symptomatic relief in contrast to omeprazole 10 mg group (odds ratio, OR: 1.89, 95% confidence interval, CI: 1.34, 2.67; p-value: 0.0005) or rabeprazole 5 mg group (OR: 2.51, 95%CI: 1.16, 5.42; p-value: 0.019); dexlansoprazole 30 mg therapy significantly improved the rate of symptomatic relief compared with rabeprazole 5 mg group (OR: 2.64, 95%CI: 1.08, 6.43; p-value: 0.03). For the rate of adverse events, there was no significant difference among all interventions.

Gastroesophageal reflux disease (GERD) is a very common benign disease of the upper gastrointestinal tract. Epidemiology surveys and relevant systematic reviews indicated the prevalence of GERD ranges 10–20% in the western countries (about 20% in USA) and 11.5% in Japan^1^,^2^,^3^. GERD could cause various esophageal, gastrointestinal, and extraesophageal symptoms (e.g., heartburn, epigastric pain and respiratory disorders), which seriously affected people’s quality of life^4^,^5^. GERD could be sorted as erosive oesophagitis (EO) and non-erosive reflux disease (NERD) based on the manifestations of esophageal mucosa damage through endoscopy and NERD is the mainstay of GERD (70%)^6^,^7^.

Proton-pump inhibitors (PPIs) have been proved as a safe and effective way to treat patients with GERD and recommended as a main acid suppressive drug by many originations’ guidelines (e.g., the European Association of Endoscopic Surgery, the American College of Gastroenterology, the Indonesian Society of Gastroenterology and Pakistan Society of Gastroenterology)^4^,^8^,^9^,^10^. The reason that PPIs could relieve the symptoms is that the drug potently decrease gastric acid secretion by inhibiting the H ion - K ion adenosine triphosphatase pump of the parietal cell^11^.

However, previous literatures focused on the comparison between PPIs and placebo^12^,^13^. Less is known about the comparisons among different PPIs (e.g., omeprazole, rabeprazole and lansoprazole). Only one indirect meta-analysis conducted a comparison between two PPIs (dexlansoprazole and esomeprazole), but the study did not provide the rank of the interventions^14^. Thus, we aimed to perform a comprehensive network meta-analyses to compare as well as rank the efficacy and safety of different PPIs in treating patients with NERD.

## Results

### Literature search

**Figure 1:**
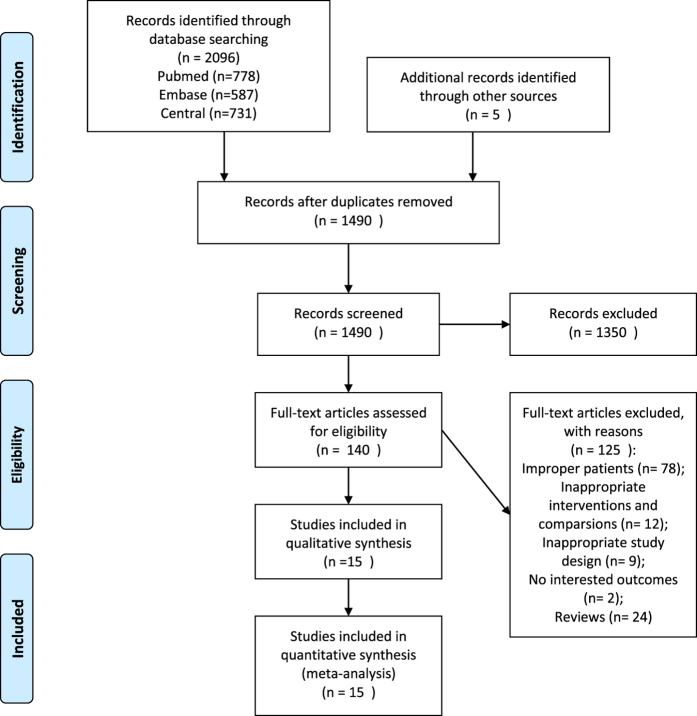
PRISMA flow diagram.

Figure 1 shows the whole process of literature searching. Initially, we imported 2101 citations into EndNote. After removing the duplicated citations, two reviewers screened 1490 titles and abstracts independently. Of these, 140 articles were potentially relevant and we reviewed full texts. We excluded 125 studies for the following reasons: improper patients, interventions and comparisons; inappropriate study design; no interested outcomes and reviews. Finally, we included 15 studies with 16 trials in the meta-analyses^15^,^16^,^17^,^18^,^19^,^20^,^21^,^22^,^23^,^24^,^25^,^26^,^27^,^28^,^29^.

### Study and patient characteristics

Table 1 shows that the publication year ranged from 1997 to 2011. Most RCTs were conducted in Europe and USA. The duration of follow-up ranged from 1 month to 6 months. The total number of patients across the studies was 6299, with an average of 394 patients per trial. The proportion of males in the included studies ranged from 28.9% to 55.8%. The positive rate of helicobacter pylori tests ranged from 13.4% to 52.7%.

### Risk of bias

**Figure 2:**
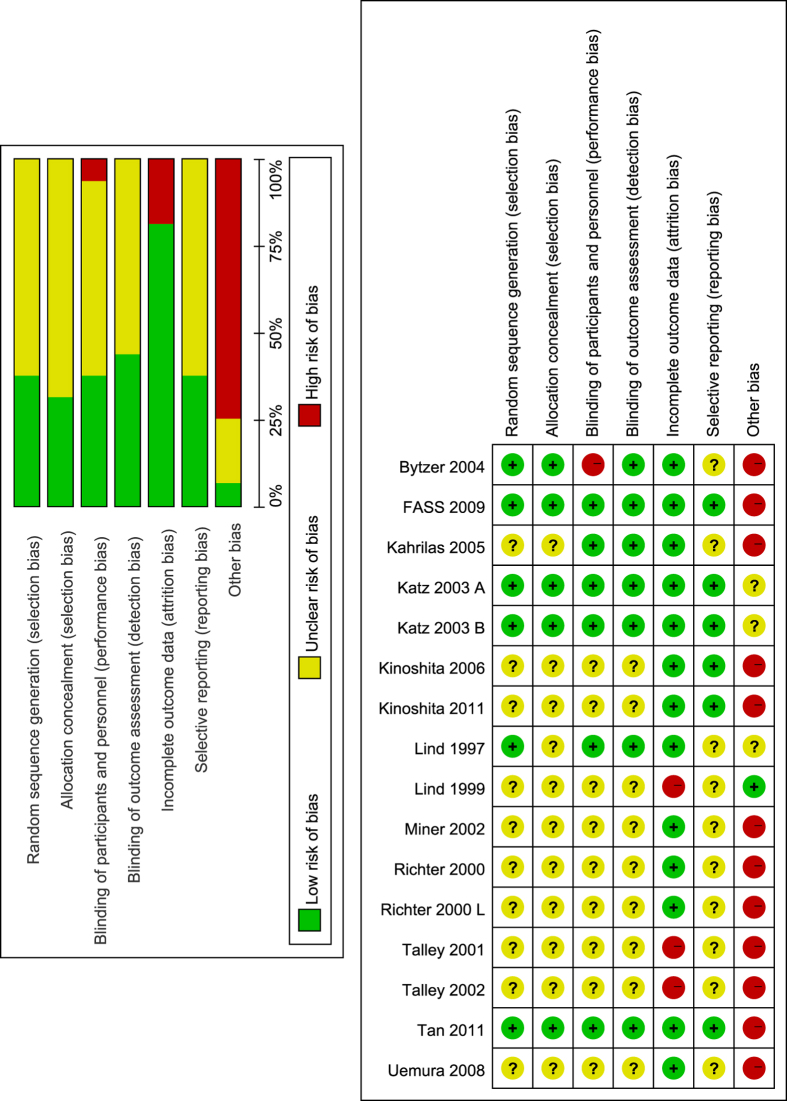
Risk of bias graph and summary. Green for low risk of bias, yellow for unclear risk of bias and red for high risk of bias. The left (risk of bias graph) shows an overall risk of bias of each domain. For example, the length of green rectangle means the number of studies being assessed as low risk of risk. The right (risk of bias summary) indicates the risk of bias of each domain in each study.

Only a small number of trials adequately described random sequence generation (37.5%) and allocation concealment (31.25%). Six trials had a low risk of bias in blinding of participants and personnel. Similarly, seven trials had a low risk of bias in blinding of outcome assessment. Most trials had a low risk of bias in incomplete outcome data (81.25%). For selective reporting domain, seven studies were judged as low risk of bias. Majority of included studies received commercial fund (75%), so they were judged as high risk of bias. All details is shown in Fig. 2.

### Rate of symptomatic relief

**Figure 3:**
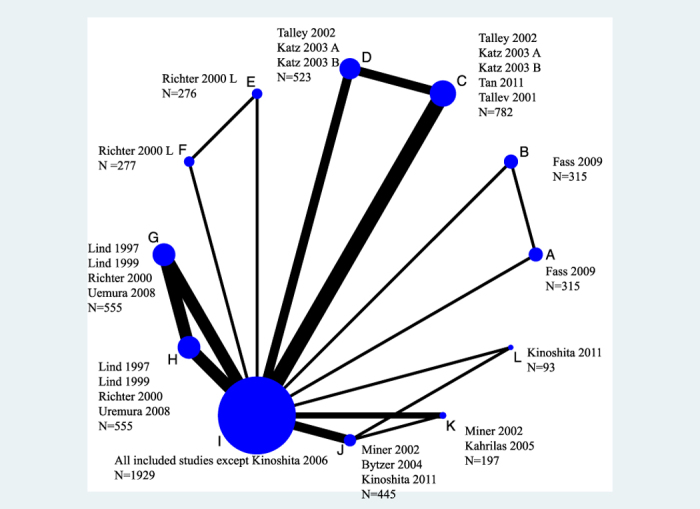
Network plot for the rate of symptomatic relief. (**A**) Dexlansoprazole 30 mg, (**B**) Dexlansoprazole 60 mg, (**C**) Esomeprazole 20 mg, (**D**) Esomeprazole 40 mg, (**E**) Lansoprazole 15 mg, (**F)** Lansoprazole 30 mg, (**G**) Omeprazole 10 mg, (**H**) Omeprazole 20 mg, (**I**) Placebo, (**J**) Rabeprazole 10 mg, (**K**) Rabeprazole 20 mg, (**L**) Rabeprazole 5 mg.

A total of 315 patients (1 trial) were assigned to dexlansoprazole 30 mg therapy, 315 (1 trial) to dexlansoprazole 60 mg therapy, 555 (4 trials) to omeprazole 10 mg therapy, 555 (4 trials) to omeprazole 20 mg therapy, 276 (1 trial) to lansoprazole 15 mg therapy, 277 (1 trial) to lansoprazole 30 mg therapy, 782 (5 trials) to esomeprazole 20 mg therapy, 523 (3 trials) to esomeprazole 40 mg therapy, 93 (1 trial) to rabeprazole 5 mg therapy, 445 (3 trials) to rabeprazole 10 mg therapy, 197 (2 trials) to rabeprazole 20 mg therapy and 1929 (15 trials) to placebo therapy. The network plot is shown in Fig. 3.

**Figure 4:**
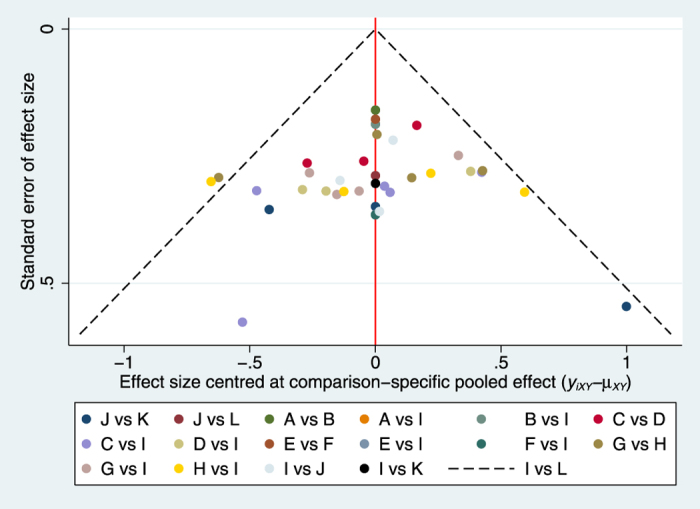
Funnel plot for the rate of symptomatic relief. (**A**) Dexlansoprazole 30 mg, (**B**) Dexlansoprazole 60 mg, (**C**) Esomeprazole 20 mg, (**D**) Esomeprazole 40 mg, (**E**) Lansoprazole 15 mg, (**F**) Lansoprazole 30 mg, (**G**) Omeprazole 10 mg, (**H**) Omeprazole 20 mg, (**I**) Placebo,(**J**) Rabeprazole 10 mg, (**K**) Rabeprazole 20 mg, (**L**) Rabeprazole 5 mg.

Compared with placebo groups, all interventions except rabeprazole 5 mg significantly increased rate of symptomatic relief. Among the comparisons of different interventions, omeprazole 20 mg group was associated with a higher rate of symptomatic relief in contrast to rabeprazole 5 mg group (OR: 2.51, 95%CI: 1.16, 5.42; p-value: 0.019) or omeprazole 10 mg group (OR: 1.89, 95%CI: 1.34, 2.67; p-value: 0.0005); dexlansoprazole 30 mg therapy significantly improved the rate of symptomatic relief compared with rabeprazole 5 mg group (OR: 2.64, 95%CI: 1.08, 6.43; p-value: 0.03). The details of all comparisons are indicated in Table 2. No inconsistency was found through global (p = 0.36) and loop-specific approach (Figure S1). Because the majority of included studies had a high risk of bias, we could not perform sensitivity analyses. To test the robustness of the results, we made meta-regressions about mean age, percentage of male and duration of follow-up. The results of meta-regressions showed that these factors had no effect on the results of network meta-analyses. The funnel plot was indicated in Fig. 4.

### Rate of adverse events

A total of 315 patients (1 trial) were assigned to dexlansoprazole 30 mg therapy, 315 (1 trial) to dexlansoprazole 60 mg therapy, 572 (5 trials) to omeprazole 10 mg therapy, 575 (5 trials) to omeprazole 20 mg therapy, 276 (1 trial) to lansoprazole 15 mg therapy, 277 (1 trial) to lansoprazole 30 mg therapy, 782 (5 trials) to esomeprazole 20 mg therapy, 523 (3 trials) to esomeprazole 40 mg therapy, 93 (1 trial) to rabeprazole 5 mg therapy, 445 (3 trials) to rabeprazole 10 mg therapy, 197 (2 trials) to rabeprazole 20 mg therapy and 1929 (15 trials) to placebo therapy. The network plot is shown in Figure S2.

**Figure 5:**
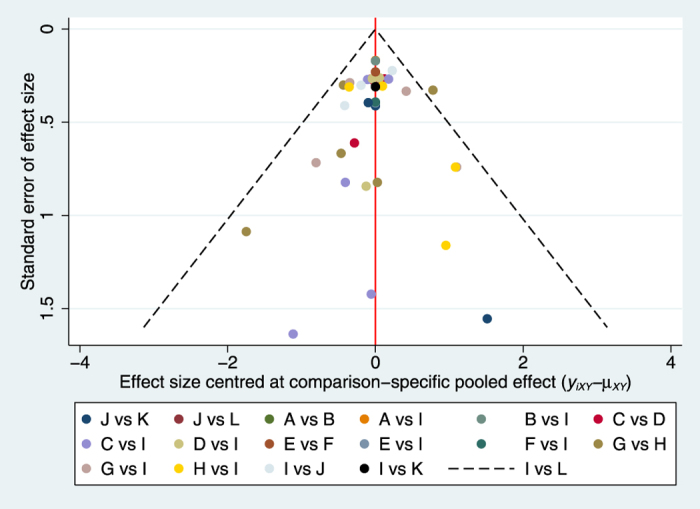
Funnel plot for the rate of adverse events. (**A**) Dexlansoprazole 30 mg, (**B**) Dexlansoprazole 60 mg, (**C**) Esomeprazole 20 mg, (D) Esomeprazole 40 mg, (**E**) Lansoprazole 15 mg, (**F**) Lansoprazole 30 mg, (**G**) Omeprazole 10 mg, (**H**) Omeprazole 20 mg, (**I**) Placebo, J: Rabeprazole 10 mg, (**K**) Rabeprazole 20 mg, (**L**) Rabeprazole 5 mg.

There was no significant difference among all interventions. The details of all comparisons is indicated in Table 3. No inconsistency was found through global (p = 0.68) and loop-specific approach (Figure S3). Because the majority of included studies had a high risk of bias, we could not perform sensitivity analyses. To test the robustness of the results, we performed meta-regressions about mean age, percentage of male and duration of follow-up. The results of meta-regressions showed they had no effect on the results of network meta-analyses. The funnel plot was indicated in Fig. 5.

### Rank of the interventions

For rate of symptomatic relief, dexlansoprazole 30 mg therapy ranked the first and placebo therapy ranked the last (Figure S4). For rate of adverse events, omeprazole 20 mg therapy had the smallest incidence and lansoprazole 30 mg therapy was associated with the highest incidence (Figure S5). The cluster plot (Figure S6) shows omeprazole 20 mg might be the best intervention in treating patients with NERD. However, we did not make a conclusion that omeprazole 20 mg is the best option because omeprazole 20 mg did not show advantage over the other interventions.

## Discussion

### Summary of the results

1. Compared with control groups, all interventions except rabeprazole 5 mg significantly increased rate of symptomatic relief. Among the comparisons of different PPIs, omeprazole 20 mg group was associated with a higher rate of symptomatic relief in contrast to rabeprazole 5 mg group or omeprazole 10 mg group; dexlansoprazole 30 mg therapy significantly improved the rate of symptomatic relief compared with rabeprazole 5 mg group; 2. For the rate of adverse events, there was no significant difference among all interventions; 3. According to overall rank results, omeprazole 20 mg might be the best intervention in treating patients with non-erosive gastroesophageal reflux.

### Comparison with previous literatures

For rate of symptomatic relief, our results were similar with previous pair-wise meta-analysis except rabeprazole 5 mg therapy^13^. The difference may be attributed to the random bias (only 1 trial with 93 patients) or the low dose (the recommended dose is 10 mg/20 mg). On the other hand, the results of previous indirect meta-analysis were different from ours^14^. Previous one included dexlansoprazole 30 mg, dexlansoprazole 60 mg, esomeprazole 40 mg and esomeprazole 20 mg. Among the four PPIs, dexlansoprazole 30 mg significantly increased symptom relief compared with esomeprazole 20 or 40 mg. However, our results showed there were no significant results between dexlansoprazole 30 mg and esomeprazole 20 or 40 mg. The possible explanation is that the sample size of our study outnumbers those of previous meta-analyses (6 studies with 2902 patents vs 3 studies with 2032 patients). For the rate of adverse events, our results were totally the same with the previous ones. Additionally, we firstly compared lansoprazole 15 mg, lansoprazole 30 mg, omeprazole 10 mg, omeprazole 10 mg, rabeprazole 10 mg, rabeprazole 20 mg and rabeprazole 5 mg.

### Limitations

Several limitations should be noted to help clinicians reasonably clarify the results. First, the sample size of one study was rather small (n = 37), which might cause potential small sample biases^20^. Thus, we performed a sensitivity analysis by excluding the study and the result remained stable. Then, there was only one study for some interventions (e.g., dexlansoprazole 30 mg therapy, dexlansoprazole 60 mg therapy, lansoprazole 15 mg therapy, lansoprazole 30 mg therapy, esomeprazole 40 mg therapy and rabeprazole 5 mg therapy) and we need to interpret these results cautiously. Additionally, we did not find study with pantoprazole for patients with NERD, which indicated our study did not comprehensively analyze all kinds of PPIs. Finally, most of included studies received funds from commercial companies, which might affect the imprecision of the results.

## Conclusions

Compared with control groups, all interventions except rabeprazole 5 mg significantly increased rate of symptomatic relief. In addition, omeprazole 20 mg group was associated with a higher rate of symptomatic relief in contrast to omeprazole 10 mg group or rabeprazole 5 mg group; dexlansoprazole 30 mg therapy significantly improved the rate of symptomatic relief in contrast to rabeprazole 5 mg group. For the rate of adverse events, there was no significant difference among all interventions. To make conclusions more reliable and applicable, future RCTs should focus on dexlansoprazole, lansoprazole and pantoprazole and be conducted in Africa, South America and Oceania.

## Materials and Methods

### Eligibility criteria

Patients: patients with NERD. The definition of NERD: patients only have the condition symptoms without the presence of oesophageal abnormalities by endoscopy.

Interventions and comparisons: We included studies which had at least two of the following interventions: placebo, dexlansoprazole, omeprazole, lansoprazole, esomeprazole, rabeprazole and pantoprazole. We did not restrict doses of the PPIs.

Outcomes: rate of symptomatic relief (heartburn) and rate of adverse events.

Study design: we included randomized clinical trials (RCTs). For cross-over studies, we used the data before wash-out period. We excluded cluster randomized clinical trials.

### Information sources and literature search

We searched PubMed, EMBASE and Cochrane library from inception until February 11, 2016. Additionally, we searched clinicaltrials.gov for the unpublished researches. Finally, we manually searched the reference lists of included studies and meta-analyses with similar topic.

### Study selection process

Initially, two authors screened the title and abstract of citations. After completing the preliminary screening, they screened potential included studies by reading their full-text. If any disagreement could not be solved by discussion, a third author was consulted.

### Data items, data abstraction process and risk of bias

Two authors abstracted study characteristics (e.g., first author, publication year and sample size), patient characteristics (e.g., mean age and gender), outcome definitions, and inclusion and exclusion criteria.

Two authors used risk of bias tool (Rob tool) to evaluate the quality of included RCTs independently^30^. The tool includes seven domains: random sequence generation, allocation concealment, blinding of participants and personnel, blinding of outcome assessment, incomplete outcome data, selective reporting and other bias (e.g., baseline imbalance, commercial funds and early drop-out). For each domain, two authors judged the quality of each RCT as high or low or unclear risk of bias.

### Data analysis

Before performing data synthesizing, we assessed the transitivity assumption and consistency assumption. For the transitivity assumption, we compared the study characters and patient characters across treatment comparisons^31^. For the consistency assumption, we use two ways to evaluate the inconsistency: global methods (the ‘design-by-treatment’ inconsistency model) and loop-specific approach^32^. Either the global P value in the global methods was less than 0.05 or the percentage of inconsistency loop was larger than 20%, we considered there was significant inconsistency in the study^33^. We conducted network meta-analyses with random model if there was a good transitivity across treatment comparisons and no significant inconsistency^34^. All data-analyses were conducted through STATA V.13.1.

If we find any significant inconsistency, we performed subgroup analyses and meta-regressions (e.g., mean age and proportion of male) to investigate the possible sources^35^. Then, we excluded studies with a high risk of bias as a form of sensitivity analysis. For the publication bias, comparison-adjusted funnel plot was used^36^. Finally, the surface under the cumulative ranking curve analysis (SUCRA) was conducted to rank all interventions. For any intervention, a larger value of SUCRA means a higher rank^36^. Additionally, we performed a cluster plot including both rate of symptomatic relief and rate of adverse events to observe the possible best intervention^36^.

## Additional Information

**How to cite this article**: Chen, L. *et al.* The efficacy and safety of proton-pump inhibitors in treating patients with non-erosive reflux disease: a network meta-analysis. *Sci. Rep.* **6**, 32126; doi: 10.1038/srep32126 (2016).

## Supplementary Material

Supplementary Information

## Figures and Tables

**Table 1:** Characteristics of included studies.

Study	Country	Comparisons	Mean age (years)	Gender(M/F)	Sample size	Duration of follow-up	Positive Helicobacter pylori test (%)
Bytzer^15^	Europe*	Rab 10 vs Pla	47/48	180/238	418	6 months	36/37
FASS^16^	USA	Dex 30 vs Dex 60 vs Pla	47.6/47.5/47.5	274/673	947	1 month	30.2/28.6/28.9
Kahrilas^17^	USA	Rab 20 vs Pla	43.1/44	85/176	261	1 month	32.6/30.3
Kinoshita^20^	Japan	Ome 10 vs Ome 20	41.7/48.2	18/19	37	1 month	29.4/30
Kinoshita^19^	Japan	Rab 5 vs Rab 10 vs Pla	46.3/46.8/49.7	128/157	285	1 month	38/42/48
Lind^21^	Denmark and Sweden	Ome 10 vs Ome 20 vs pla	49/50/51	206/303	509	1 month	/
Lind^22^	Denmark and Sweden	Ome 10 vs Ome 20 vs pla	51/52/48	179/245	424	6 months	/
Miner^23^	USA	Rab 10 vs Rab 20 vs Pla	44.4/45.5/46.1	77/126	203	1 month	26.6/35.3/39.7
Richter^24^	USA	Ome 10 vs Ome 20 vs pla	50/49.5/49.7	193/166	359	1 month	/
Richter^25^ L	USA	Lan 15 vs Lan 30 vs Pla	44.9/45.1/46.3	257/363	620	2 months	23.9/23.8/13.4
Talley^26^	Denmark, Finland, Norway and Sweden	Eso 20 vs Pla	49/49	191/151	342	6 months	38/33
Talley^27^	UK	Eso 20 vs Eso 40 vs Pla	48.4/48/48.2	328/393	721	6 months	35.8/30.7/39
Tan^28^	China	Eso 20 vs Pla	48.5/48.3	62/113	175	2 months	/
Uemura^29^	Japan	Ome 10 vs Ome 20 vs pla	44.4/43.8/42.4	143/138	281	1 month	38.5/52.7/45.7
Katz^18^ A	USA	Eso 20 vs Eso 40 vs Pla	47/47/46	143/225	368	1 month	37/39/29
Katz^18^ B	USA	Eso 20 vs Eso 40 vs Pla	46/45/47	126/223	349	1 month	43/30/38

Rab 5: Rabeprazole 5 mg; Rab 10: Rabeprazole 10 mg; Rab 20: Rabeprazole 20 mg; Pla: Placebo; Dex 30: Dexlansoprazole 30 mg; Dex 60: Dexlansoprazole 60 mg; Ome 10: Omeprazole 10 mg; Ome 20: Omeprazole 20 mg; Lan 15: Lansoprazole 15 mg; Lan 30: Lansoprazole 30 mg; Eso 20: Esomeprazole 20 mg; Eso 40: Esomeprazole 40 mg.

^*^Greece, Italy, the Netherlands, Spain, France, Portugal, Sweden, Denmark, Ireland, Belgium, United Kingdom, Russia, Poland, and Lithuania.

**Table 2:** Results of pairwise and network meta-analyses for the rate of symptomatic relief.

Dex30	1.23(0.9, 1.68);p-value: 0.2	NA	NA	NA	NA	NA	NA	**5.09(3.53, 7.35);p-value < 0.00001**	NA	NA	NA
1.23(0.71,2.13);p-value: 0.46	Dex60	NA	NA	NA	NA	NA	NA	**4.15(2.88, 6);p-value** < **0.00001**	NA	NA	NA
1.48(0.75,2.95);p-value: 0.26	1.21(0.61,2.41);p-value: 0.59	Eso20	0.89(0.68, 1.15);p-value: 0.37	NA	NA	NA	NA	**3.46(2.46, 3.87);p-value** < **0.00001**	NA	NA	NA
1.38(0.68,2.81);p-value: 0.69	1.13(0.55,2.29);p-value: 0.74	0.93(0.65,1.34);p-value: 0.69	Eso40	NA	NA	NA	NA	**3.55(2.32, 5.44);p-value** < **0.00001**	NA	NA	NA
1.52(0.54,4.26);p-value: 0.43	1.24(0.44,3.48);p-value: 0.68	1.03(0.41,2.58);p-value: 0.95	1.10(0.43,2.82);p-value: 0.84	Lan15	1.09(0.77, 1.54);p-value: 0.63	NA	NA	**3.34(1.63, 6.83);p-value:0.0009**	NA	NA	NA
1.66(0.59,4.64);p-value: 0.34	1.35(0.48,3.79);p-value: 0.57	1.12(0.44,2.81);p-value: 0.81	1.20(0.47,3.07);p-value: 0.7	1.09(0.61,1.93);p-value: 0.77	Lan30	NA	NA	**3.07(1.5, 6.28);p-value: 0.002**	NA	NA	NA
1.99(1.00,3.96);p-value: 0.05	1.62(0.81,3.23);p-value: 0.17	1.34(0.80,2.23);p-value: 0.27	1.44(0.84,2.48);p-value: 0.18	1.30(0.52,3.28);p-value: 0.57	1.20(0.48,3.02);p-value: 0.7	Ome10	0.36(0.16, 0.81);p-value: 0.01	**2.52(1.9, 3.35);p-value** < **0.00001**	NA	NA	NA
1.05(0.53,2.10);p-value: 0.89	0.86(0.43,1.71);p-value: 0.67	0.71(0.42,1.19);p-value: 0.2	0.76(0.44,1.32);p-value: 0.33	0.69(0.27,1.74);p-value: 0.44	0.63(0.25,1.60);p-value: 0.33	**0.53(0.37,0.75);p-value: 0.0005**	Ome20	**4.93(2.94, 8.26);p-value** < **0.00001**	NA	NA	NA
**5.09(2.84,9.13);p-value** < **0.00001**	**4.15(2.32,7.45);p-value** < **0.00001**	**3.43(2.39,4.93);p-value** < **0.00001**	**3.69(2.46,5.53);p-value** < **0.00001**	**3.34(1.43,7.80);p-value** < **0.0053**	**3.07(1.32,7.17);p-value** < **0.0092**	**2.56(1.78,3.69);p-value** < **0.00001**	**4.85(3.34,7.04);p-value** < **0.00001**	Pla	**0.38**(0.28, 0.51);**p-value** < **0.00001**	**0.19(0.05, 0.78);p-value: 0.02**	0.58(0.32, 1.04);p-value: 0.07
1.80(0.88,3.69);p-value: 0.11	1.47(0.72,3.01);p-value: 0.31	1.22(0.70,2.10);p-value: 0.48	1.31(0.74,2.33);p-value: 0.35	1.18(0.46,3.04);p-value: 0.73	1.09(0.42,2.79);p-value: 0.86	0.91(0.52,1.57);p-value: 0.74	1.72(0.99,2.99);p-value: 0.054	**0.35(0.24,0.53);p-value** < **0.00001**	Rab10	0.98(0.49, 1.94);p-value: 0.95	1.33(0.76, 2.34);p-value: 0.32
1.23(0.52,2.91);p-value: 0.64	1.00(0.42,2.38);p-value: 1	0.83(0.40,1.71);p-value: 0.62	0.89(0.42,1.89);p-value: 0.76	0.81(0.28,2.33);p-value: 0.7	0.74(0.26,2.14);p-value: 0.57	0.62(0.30,1.28);p-value: 0.2	1.17(0.56,2.44);p-value: 0.68	**0.24(0.13,0.46);p-value** < **0.00001**	0.68(0.35,1.32);p-value: 0.26	Rab20	NA
**2.64(1.08,6.43);p-value: 0.03**	2.15(0.88,5.25);p-value: 0.09	1.78(0.83,3.81);p-value: 0.14	1.91(0.87,4.19);p-value: 0.11	1.73(0.59,5.11);p-value: 0.32	1.59(0.54,4.70);p-value: 0.4	1.33(0.62,2.85);p-value: 0.46	**2.51(1.16,5.42);p-value: 0.019**	0.52(0.26,1.02);p-value: 0.064	1.46(0.75,2.84);p-value: 0.27	2.14(0.88,5.20);p-value: 0.093	Rab5

Results of pairwise meta-analyses were listed in right upper triangles and results of network meta-analyses were listed in left lower triangles. Rab 5: Rabeprazole 5 mg; Rab 10: Rabeprazole 10 mg; Rab 20: Rabeprazole 20 mg; Pla: Placebo; Dex 30: Dexlansoprazole 30 mg; Dex 60: Dexlansoprazole 60 mg; Ome 10: Omeprazole 10 mg; Ome 20: Omeprazole 20 mg; Lan 15: Lansoprazole 15 mg; Lan 30: Lansoprazole 30 mg; Eso 20: Esomeprazole 20 mg; Eso 40: Esomeprazole 40 mg. The number which was painted by a style of overstriking indicated there was a significant difference between two treatments.

**Table 3:** Results of pairwise and network meta-analyses for the rate of adverse events.

Dex 30	1.15(0.83, 1.61);p-value: 0.4	NA	NA	NA	NA	NA	NA	1.15(0.82, 1.6);p-value: 0.41	NA	NA	NA
1.15(0.65,2.04);p-value: 0.63	Dex 60	NA	NA	NA	NA	NA	NA	0.99(0.71, 1.39);p-value: 0.98	NA	NA	NA
1.11(0.53,2.33);p-value: 0.78	0.97(0.46,2.02);p-value: 0.94	Eso 20	0.87(0.61, 1.24);p-value: 45	NA	NA	NA	NA	1(0.7, 1.43);p-value:0.99	NA	NA	NA
1.00(0.48,2.08);p-value: 1	0.86(0.41,1.81);p-value: 0.69	0.89(0.56,1.42);p-value: 0.62	Eso 40	NA	NA	NA	NA	1.14(0.8, 1.64);p-value: 0.46	NA	NA	NA
1.02(0.35,2.97);p-value: 0.97	0.88(0.30,2.58);p-value: 0.82	0.92(0.33,2.54);p-value: 0.87	1.02(0.37,2.84);p-value: 0.97	Lan 15	0.79(0.5, 1.24);p-value: 0.31	NA	NA	1.12(0.52, 2.44);p-value: 0.77	NA	NA	NA
0.81(0.28,2.34);p-value: 0.7	0.70(0.24,2.03);p-value: 0.51	0.73(0.26,1.99);p-value: 0.55	0.81(0.30,2.23);p-value: 0.68	0.79(0.41,1.51);p-value: 0.48	Lan 30	NA	NA	1.42(0.66, 3.05);p-value: 0.37	NA	NA	NA
1.23(0.58,2.58);p-value: 0.59	1.06(0.51,2.24);p-value: 0.88	1.10(0.56,2.16);p-value: 0.78	1.23(0.63,2.41);p-value: 0.54	1.20(0.43,3.34);p-value: 0.73	1.52(0.55,4.19);p-value: 0.42	Ome 10	1.23(0.57, 2.65);p-value: 0.6	0.88(0.45, 1.69);p-value: 0.69	NA	NA	NA
1.37(0.64,2.92);p-value: 0.42	1.18(0.55,2.54);p-value: 0.67	1.23(0.61,2.46);p-value: 0.56	1.37(0.69,2.74);p-value: 0.37	1.34(0.48,3.77);p-value: 0.58	1.69(0.60,4.72);p-value: 0.32	1.11(0.69,1.80);p-value: 0.67	Ome 20	0.82(0.48, 1.4);p-value: 0.46	NA	NA	NA
1.15(0.65,2.03);p-value: 0.63	0.99(0.56,1.76);p-value: 0.97	1.03(0.65,1.64);p-value: 0.9	1.15(0.72,1.84);p-value: 0.56	1.12(0.45,2.78);p-value: 0.81	1.42(0.58,3.48);p-value: 0.44	0.94(0.58,1.51);p-value: 0.8	0.84(0.51,1.39);p-value: 0.49	Pla	0.79(0.55, 1.55);p-value: 0.22	0.86(0.39, 1.92);p-value: 0.72	1.03(0.56, 1.9);p-value: 0.91
0.92(0.45,1.88);p-value: 0.82	0.80(0.39,1.63);p-value: 0.54	0.83(0.43,1.57);p-value: 0.58	0.92(0.49,1.75);p-value: 0.8	0.90(0.33,2.46);p-value: 0.84	1.14(0.42,3.08);p-value: 0.8	0.75(0.39,1.42);p-value: 0.39	0.67(0.35,1.29);p-value: 0.23	0.80(0.52,1.24);p-value: 0.31	Rab 10	0.82(0.37, 1.85);p-value: 0.64	1.1(0.61, 1.99);p-value: 0.75
0.85(0.32,2.24);p-value: 0.74	0.73(0.28,1.94);p-value: 0.52	0.76(0.30,1.90);p-value: 0.56	0.85(0.34,2.13);p-value: 0.73	0.83(0.25,2.75);p-value: 0.76	1.05(0.32,3.45);p-value: 0.94	0.69(0.27,1.74);p-value: 0.44	0.62(0.24,1.59);p-value: 0.32	0.74(0.33,1.62);p-value: 0.46	0.92(0.41,2.07);p-value: 0.84	Rab 20	NA
1.09(0.44,2.69);p-value: 0.85	0.95(0.38,2.33);p-value: 0.91	0.98(0.42,2.27);p-value: 0.96	1.10(0.47,2.54);p-value: 0.83	1.07(0.34,3.35);p-value: 0.91	1.35(0.43,4.20);p-value: 0.61	0.89(0.38,2.06);p-value: 0.79	0.80(0.34,1.88);p-value: 0.61	0.95(0.48,1.91);p-value: 0.88	1.19(0.60,2.37);p-value: 0.62	1.29(0.47,3.55);p-value: 0.62	Rab 5

Results of pairwise meta-analyses were listed in right upper triangles and results of network meta-analyses were listed in left lower triangles. Rab 5: Rabeprazole 5 mg; Rab 10: Rabeprazole 10 mg; Rab 20: Rabeprazole 20 mg; Pla: Placebo; Dex 30: Dexlansoprazole 30 mg; Dex 60: Dexlansoprazole 60 mg; Ome 10: Omeprazole 10 mg; Ome 20: Omeprazole 20 mg; Lan 15: Lansoprazole 15 mg; Lan 30: Lansoprazole 30 mg; Eso 20: Esomeprazole 20 mg; Eso 40: Esomeprazole 40 mg.
