# Supplementary material for: The efficacy and safety of proton-pump inhibitors in treating patients with non-erosive reflux disease: a network meta-analysis
Source: Sci Rep. 2016 Sep 1;6:32126. doi: 10.1038/srep32126 (PMC5007473; doi:10.1038/srep32126)
Supplement: Supplementary Information [file srep32126-s1.pdf]

The efficacy and safety of proton-pump inhibitors in treating patients with non-erosive reflux disease: a network meta-analysis

Lingxiao Chen[1], MD; Yujie Chen[2], MD; Bo Li[3], MD

Lingxiao Chen and Yujie Chen contributed equally.

1. Tianjin Medical University, Tianjin, China.

2. Department of Oncology, Tianjin Medical University General Hospital, Tianjin, China.

3. Department of Gastroenterology, China Academy of Chinese Medical Sciences Xiyuan Hospital, Beijing, China.

Correspondence to: Dr Bo Li, associate chief physician, China Academy of Chinese Medical Sciences Xiyuan Hospital, Beijing (100091), China. Email: dr.libo@vip.163.com

SUPPORTING INFORMATION

Additional Supporting Information may be found in the online version of this article:

Figure S1. Inconsistency test based on loop-specific approach for rate of symptomatic relief. A: Dexlansoprazole 30mg, B: Dexlansoprazole 60mg, C: Esomeprazole 20mg, D: Esomeprazole 40mg, E: Lansoprazole 15mg, F: Lansoprazole 30mg, G: Omeprazole 10mg, H: Omeprazole 20mg, I: Placebo, J: Rabeprazole 10mg, K: Rabeprazole 20mg, L: Rabeprazole 5mg.

Figure S2. Network plot for rate of adverse events. A: Dexlansoprazole 30mg, B: Dexlansoprazole 60mg, C: Esomeprazole 20mg, D: Esomeprazole 40mg, E: Lansoprazole 15mg, F: Lansoprazole 30mg, G: Omeprazole 10mg, H: Omeprazole 20mg, I: Placebo, J: Rabeprazole 10mg, K: Rabeprazole 20mg, L: Rabeprazole 5mg.

Figure S3. Inconsistency test based on loop-specific approach for rate of adverse events. A: Dexlansoprazole 30mg, B: Dexlansoprazole 60mg, C: Esomeprazole 20mg, D: Esomeprazole 40mg, E: Lansoprazole 15mg, F: Lansoprazole 30mg, G: Omeprazole 10mg, H: Omeprazole 20mg, I: Placebo, J: Rabeprazole 10mg, K: Rabeprazole 20mg, L: Rabeprazole 5mg.

Figure S4. SUCRA rank plot for rate of symptomatic relief. Rab 5: Rabeprazole 5mg; Rab 10: Rabeprazole 10mg; Rab 20: Rabeprazole 20mg; Pla: Placebo; Dex 30: Dexlansoprazole 30mg; Dex 60: Dexlansoprazole 60mg; Ome 10: Omeprazole 10mg; Ome 20: Omeprazole 20mg; Lan 15: Lansoprazole 15mg; Lan 30: Lansoprazole 30mg; Eso 20: Esomeprazole 20mg; Eso 40: Esomeprazole 40mg.

Figure S5. SUCRA rank plot for rate of adverse events. Rab 5: Rabeprazole 5mg; Rab

10: Rabeprazole 10mg; Rab 20: Rabeprazole 20mg; Pla: Placebo; Dex 30: Dexlansoprazole 30mg; Dex 60: Dexlansoprazole 60mg; Ome 10: Omeprazole 10mg; Ome 20: Omeprazole 20mg; Lan 15: Lansoprazole 15mg; Lan 30: Lansoprazole 30mg; Eso 20: Esomeprazole 20mg; Eso 40: Esomeprazole 40mg.

Figure S6. Cluster plot combing with rate of symptomatic relief and rate of adverse events. Rab 5: Rabeprazole 5mg; Rab 10: Rabeprazole 10mg; Rab 20: Rabeprazole 20mg; Pla: Placebo; Dex 30: Dexlansoprazole 30mg; Dex 60: Dexlansoprazole 60mg; Ome 10: Omeprazole 10mg; Ome 20: Omeprazole 20mg; Lan 15: Lansoprazole 15mg; Lan 30: Lansoprazole 30mg; Eso 20: Esomeprazole 20mg; Eso 40: Esomeprazole 40mg.

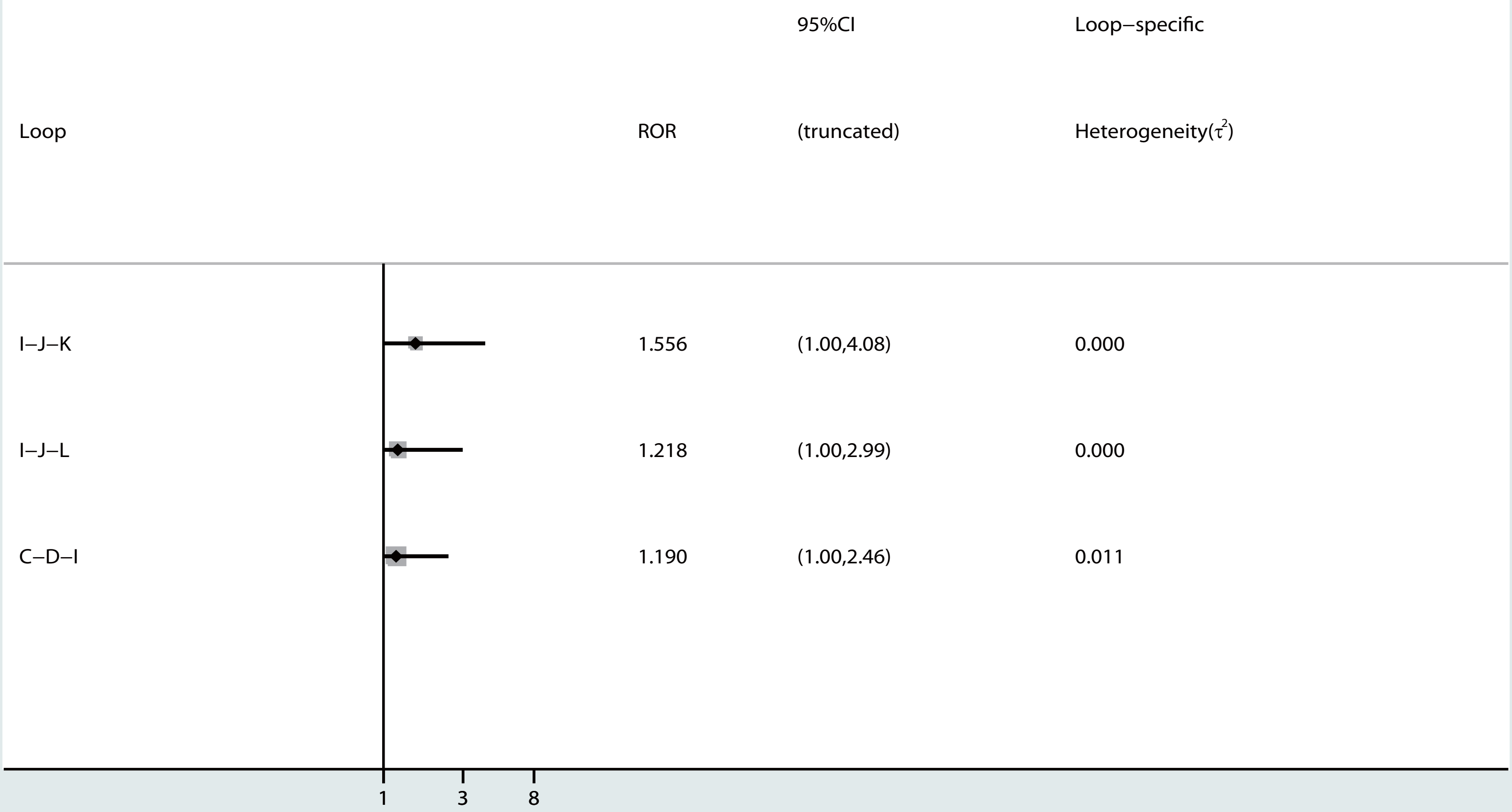

*** Loop(s) [A−B−I] [E−F−I] [G−H−I] are formed only by multi−arm trial(s) − Consistent by definition

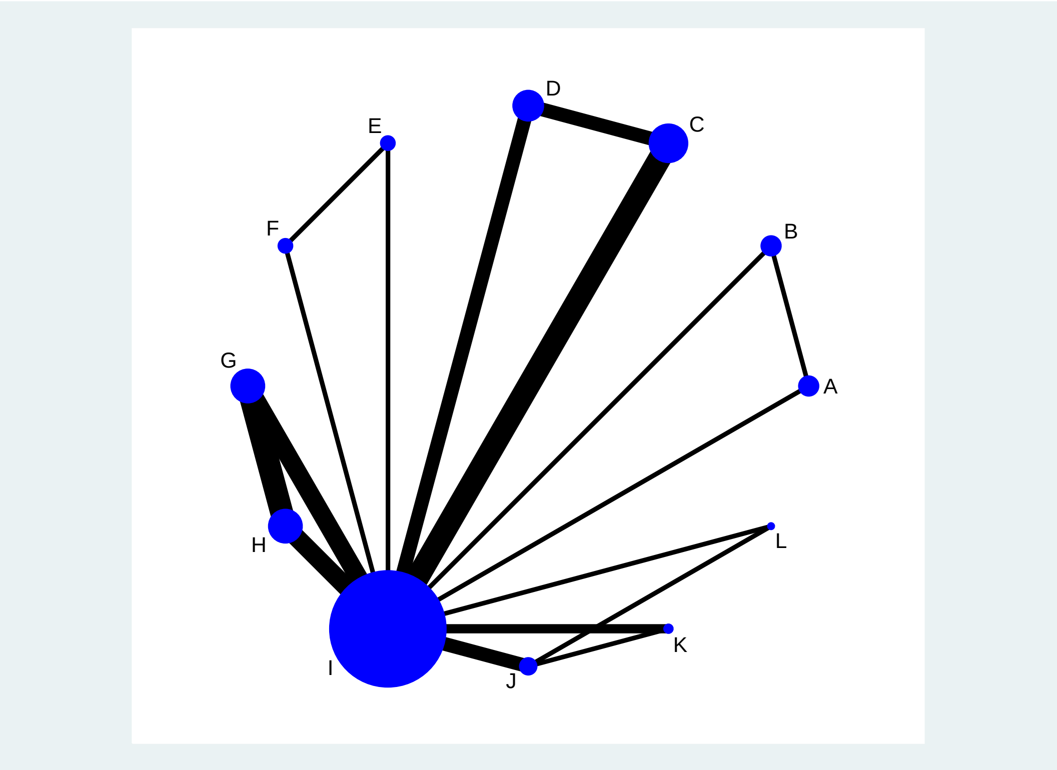
D
C
E
F
B
G
A
H
L
I
K
J

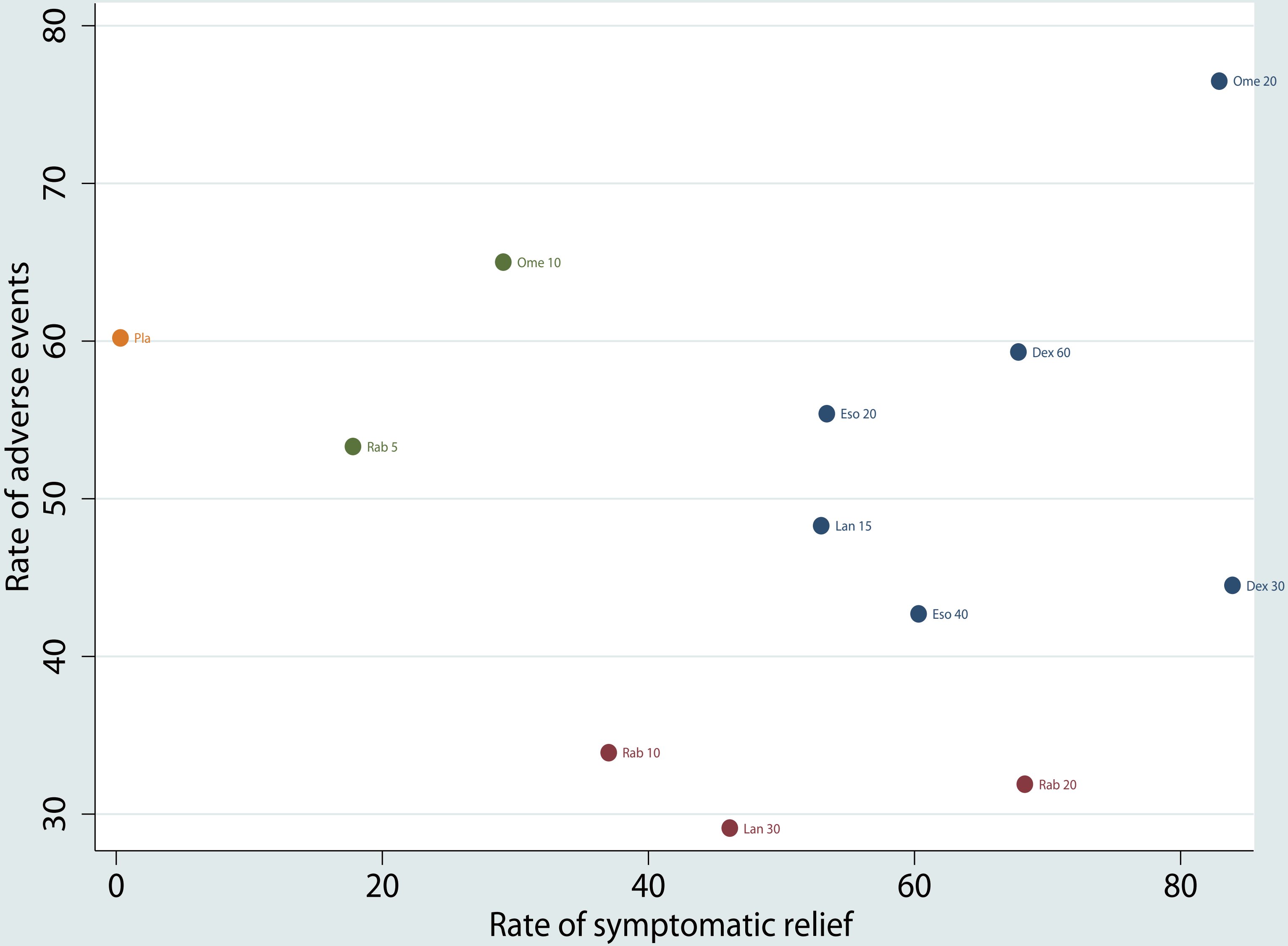
Ome 20
Ome 10
Pla
Dex 60
Eso 20
Rab 5
Lan 15
Dex 30
Eso 40
Rab 10
Rab 20
Lan 30
80
70
60
50
40
30
0
20
40
60
80
Rate of adverse events
Rate of symptomatic relief

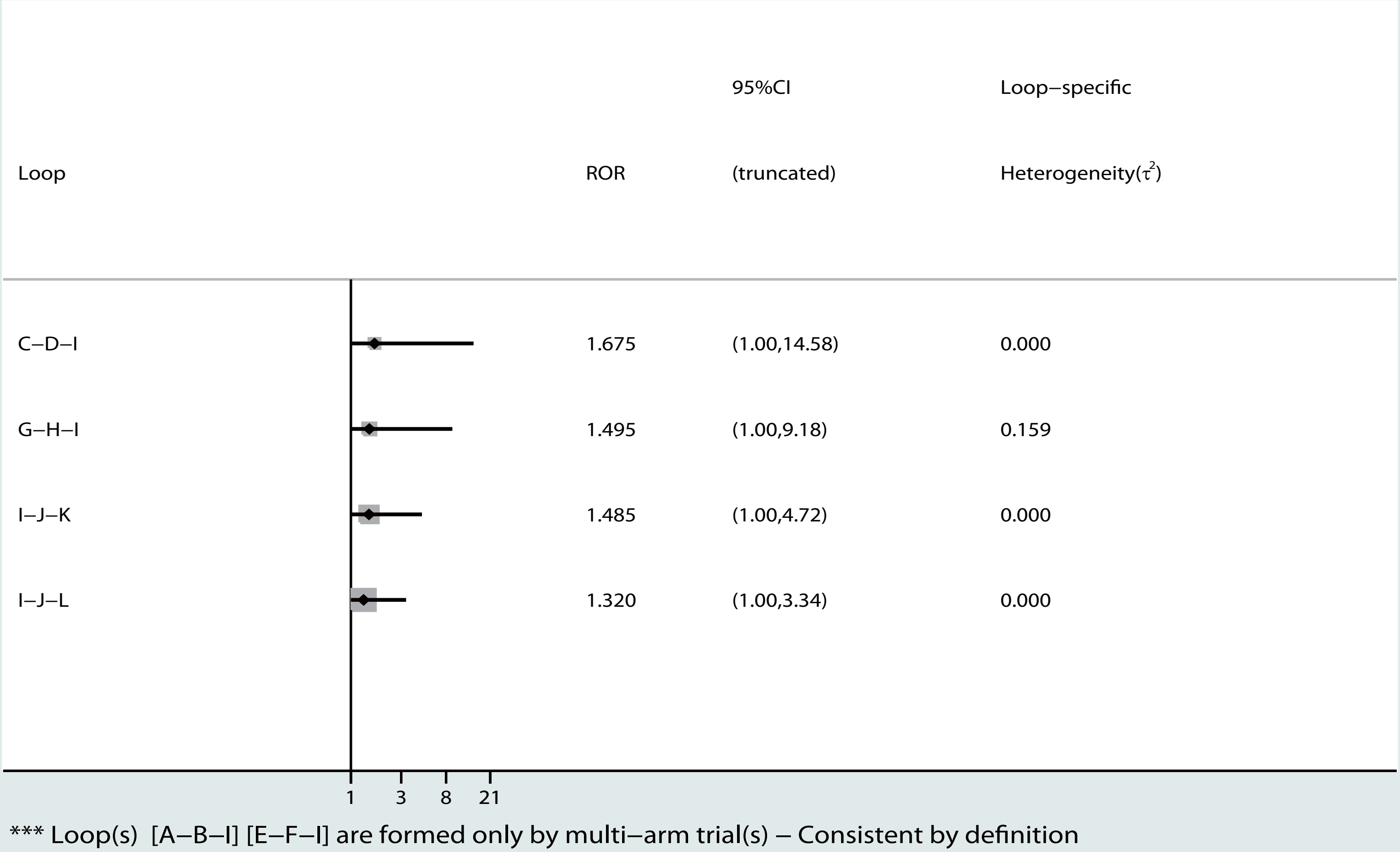

Loop
ROR
95%CI (truncated)
Loop−specific Heterogeneity(τ^2)
C−D−I
1.675
(1.00,14.58)
0.000
G−H−I
1.495
(1.00,9.18)
0.159
I−J−K
1.485
(1.00,4.72)
0.000
I−J−L
1.320
(1.00,3.34)
0.000
1
3
8
21
*** Loop(s) [A−B−I] [E−F−I] are formed only by multi−arm trial(s) − Consistent by definition

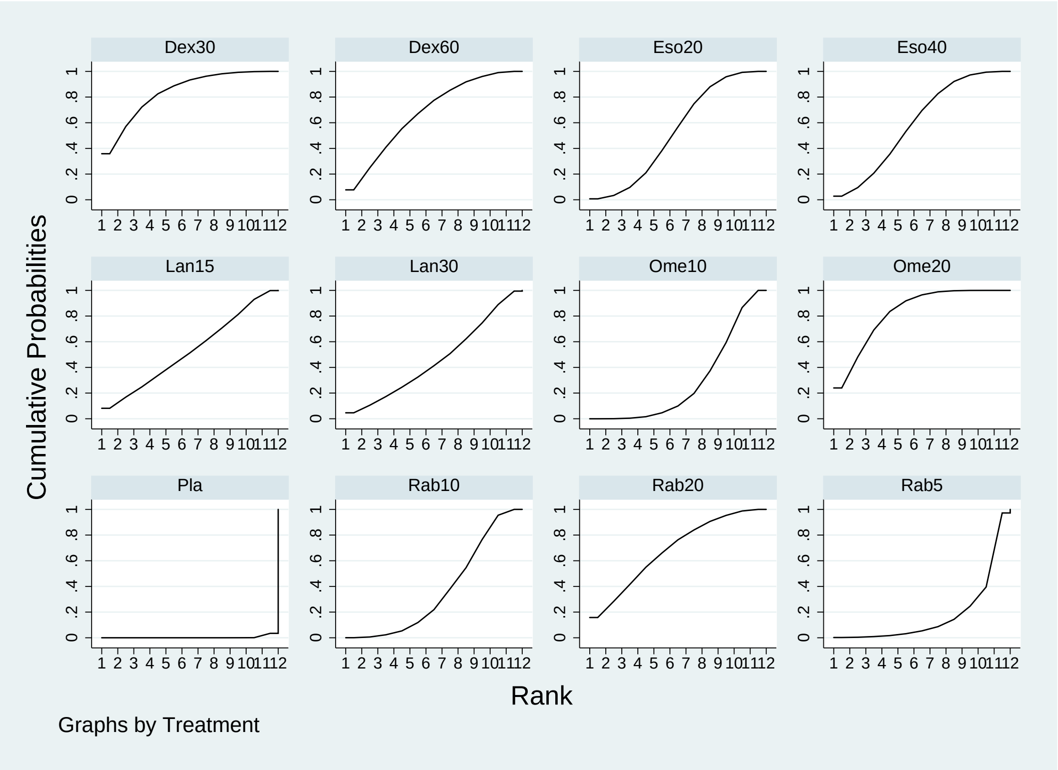

Dex30
Dex60
Eso20
Eso40
Lan15
Lan30
Ome10
Ome20
Pla
Rab10
Rab20
Rab5
0 .2 .4 .6 .8 1
1 2 3 4 5 6 7 8 9 10 11 12
Cumulative Probabilities
Rank
Graphs by Treatment

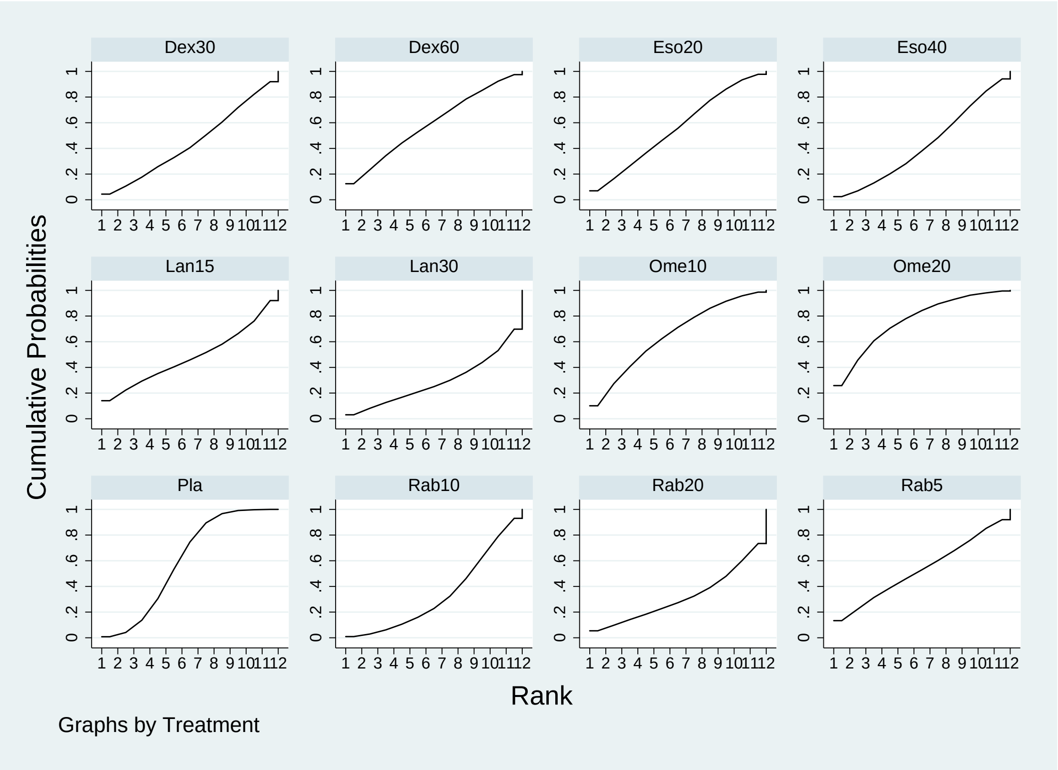
Dex30
Dex60
Eso20
Eso40
Lan15
Lan30
Ome10
Ome20
Pla
Rab10
Rab20
Rab5
Cumulative Probabilities
Rank
Graphs by Treatment
